# Confounds in neuroimaging: A clear case of sex as a confound in brain-based prediction

**DOI:** 10.3389/fneur.2022.960760

**Published:** 2022-12-19

**Authors:** Kenneth A. Weber, Zachary M. Teplin, Tor D. Wager, Christine S. W. Law, Nitin K. Prabhakar, Yoni K. Ashar, Gadi Gilam, Suchandrima Banerjee, Scott L. Delp, Gary H. Glover, Trevor J. Hastie, Sean Mackey

**Affiliations:** ^1^Systems Neuroscience and Pain Lab, Department of Anesthesiology, Perioperative and Pain Medicine, Stanford University School of Medicine, Palo Alto, CA, United States; ^2^Department of Psychological and Brain Sciences, Dartmouth College, Hanover, NH, United States; ^3^Division of Physical Medicine and Rehabilitation, Department of Orthopaedic Surgery, Stanford University School of Medicine, Palo Alto, CA, United States; ^4^Department of Psychiatry, Weill Cornell Medicine, New York, NY, United States; ^5^The Institute of Biomedical and Oral Research, Faculty of Dental Medicine, Hebrew University of Jerusalem, Jerusalem, Israel; ^6^General Electric Healthcare, Chicago, IL, United States; ^7^Department of Bioengineering and Mechanical Engineering, Stanford University, Palo Alto, CA, United States; ^8^Radiological Sciences Laboratory, Department of Radiology, Stanford University School of Medicine, Palo Alto, CA, United States; ^9^Department of Statistics, Stanford University, Palo Alto, CA, United States

**Keywords:** neuroimaging, magnetic resonance imaging, brain, muscle strength, sex, machine learning, confounding variables, biomarkers

## Abstract

Muscle weakness is common in many neurological, neuromuscular, and musculoskeletal conditions. Muscle size only partially explains muscle strength as adaptions within the nervous system also contribute to strength. Brain-based biomarkers of neuromuscular function could provide diagnostic, prognostic, and predictive value in treating these disorders. Therefore, we sought to characterize and quantify the brain's contribution to strength by developing multimodal MRI pipelines to predict grip strength. However, the prediction of strength was not straightforward, and we present a case of sex being a clear confound in brain decoding analyses. While each MRI modality—structural MRI (i.e., gray matter morphometry), diffusion MRI (i.e., white matter fractional anisotropy), resting state functional MRI (i.e., functional connectivity), and task-evoked functional MRI (i.e., left or right hand motor task activation)—and a multimodal prediction pipeline demonstrated significant predictive power for strength (*R*^2^ = 0.108–0.536, *p* ≤ 0.001), after correcting for sex, the predictive power was substantially reduced (*R*^2^ = −0.038–0.075). Next, we flipped the analysis and demonstrated that each MRI modality and a multimodal prediction pipeline could significantly predict sex (accuracy = 68.0%−93.3%, AUC = 0.780–0.982, *p* < 0.001). However, correcting the brain features for strength reduced the accuracy for predicting sex (accuracy = 57.3%−69.3%, AUC = 0.615–0.780). Here we demonstrate the effects of sex-correlated confounds in brain-based predictive models across multiple brain MRI modalities for both regression and classification models. We discuss implications of confounds in predictive modeling and the development of brain-based MRI biomarkers, as well as possible strategies to overcome these barriers.

## Introduction

The neuromuscular system is highly adaptable. Given appropriate training, muscle strength can increase to meet the physical demands placed on the body. Morphometric adaptations associated with strength training include changes in the contractile elements of the muscles and the non-contractile tissues—the most visible being the increase in muscle size, muscle hypertrophy, which typically accompanies gains in strength (1). Strength, however, is only partially explained by the structural and architectural properties of the muscles. Adaptions within the central nervous system are understood to be a factor in force generation (2). Early gains in strength can precede any evidence of hypertrophy, and strength training in one limb can increase strength in the contralateral limb (3). Neural adaptations may increase force generation through improved intra- and inter-muscular coordination or more complete activation of the motoneuron pool. Thus, in addition to the muscles, the brain plays a role in strength as well (2).

Magnetic resonance imaging (MRI) has become a key tool for the non-invasive mapping of the human brain. With MRI, we can quantitatively characterize both structural and functional properties of the brain. From T_1_-weighted and T_2_-weighted structural imaging, the morphometry of the cortical and subcortical gray matter can be measured. Diffusion-weighted imaging can probe the integrity of the white matter tracts connecting gray matter regions. With functional MRI, dynamic fluctuations in neural signaling can be extracted to assess network-level neural processing across the brain and identify brain regions activated during experimental tasks. Together these complementary measures provide a valuable multimodal macroscale representation of the human brain.

Multivariate predictive modeling and machine-learning techniques are increasingly being adopted in the brain MRI field to develop and implement brain-based biomarkers of health and disease. These models are beginning to show promise in extracting patterns of information from the high-dimensional set of brain features to make predictions across individuals. A biomarker, broadly defined, is an indicator of normal biological processes, pathogenic processes, or responses to therapeutic intervention (4). Muscle weakness is a common finding in many neurological, neuromuscular, and musculoskeletal diseases. The pattern of weakness can help localize the site of pathology or injury and inform care; however, when weakness is identified, the contributing component, muscular or neural, may not be clear. A valid brain-based MRI biomarker of strength could be helpful for clinical and research communities in several ways: (1) prognosis (i.e., for indicating the likely progression of health or disease; (2) identifying patients likely to respond to a particular treatment (i.e., prediction); (3) identifying a specific disorder based on the pattern of brain pathology (i.e., diagnosis); and (4) identifying targets for therapeutic intervention (4).

However, the interpretation of multivariate prediction models can be problematic in the presence of confounds—variables that are not of direct interest but correlated with the predicted variable (5–7). Confounds create ambiguity in the source of the information driving the prediction. While a model may accurately predict a measure, the neurobiological information driving the prediction could be related to the confound and not the target measure itself. We sought to use multimodal brain MRI and grip strength measures to characterize and quantify the brain's contribution to strength through multivariate predictive modeling. While each MRI modality and a multimodal model significantly predicted strength in an independent testing dataset, the prediction of strength was not straightforward. Here we present a case of sex being a clear confound in the brain decoding analyses, obfuscating the relationship between the brain features and strength and creating ambiguity in the interpretation of the measure being predicted: strength or sex.

## Materials and methods

### Dataset

Multimodal 3T brain MRI datasets were acquired from the Washington University, University of Minnesota, and Oxford University (WU-Minn) Human Connectome Project (HCP) 1,200 subjects release, which contains structural MRI (T_1_-weighted and T_2_-weighted), diffusion MRI, resting state fMRI, and task fMRI in healthy young adults (8). For the 3T datasets, imaging was performed supine in 1,113 participants (average age ± one standard deviation = 28.8 ± 3.7 years, 606 females) using a Siemens 3T Connectome Skyra magnetic resonance scanner (location Washington University, St. Louis, MO, USA) equipped with a standard 32-channel Siemens receive head coil and a specially designed Siemens body transmission coil to accommodate the specialized gradients of the connectome scanners (56 cm bore, maximum gradient strength 100 mT/m). Healthy was defined broadly in the HCP to be representative of the variability in behavior, ethnicity, and socioeconomic status of the population. The exclusion criteria were limited to diabetes, hypertension, severe neurodevelopmental disorders, and diagnosed neuropsychiatric disorders and neurological conditions. The HCP has released preprocessed datasets that follow the HCP standard processing pipelines (9). The HCP preprocessed images were used as inputs into the analyses. Any processing steps in addition to the standard HCP pipeline are described in the corresponding sections.

### Gray matter features

The HCP preprocessing pipeline for the T_1_-weighted and T_2_-weighted structural images included averaging of the T_1_-weighted or T_2_-weighted images if multiple images were collected; distortion correction; alignment with MNI152 space; bias field correction; and spatial normalization to 0.7 mm^3^ MNI152 space. The processed T_1_-weighted image is considered the native volume space for each subject. In preprocessing, the T_2_-weighted image and processed diffusion MRI and fMRI datasets were registered to the T_1_-weighted space prior to normalization to MNI152 space. Cortical (i.e., pial and white matter surfaces) and subcortical regions were automatically segmented with FreeSurfer (version = 5.2) using both the T_1_-weighted and T_2_-weighted images to estimate the cerebral cortical ribbon (10). The extracted and tabulated FreeSurfer measures included in the HCP 1,200 subjects release were used as the features in the gray matter prediction pipelines. The features included 68 cortical thickness and 68 cortical area measures from 34 regions (17 left and 17 right regions) based on the Desikan-Killiany cortical atlas and subcortical volumes from 19 regions (nine left and nine right subcortical regions and one midline brainstem region) totaling 155 gray matter features (Supplementary Table 1) (11, 12).

### White matter features

The HCP preprocessing pipeline for diffusion MRI included *b*_0_ image intensity normalization, distortion correction, eddy-current correction, motion correction, gradient non-linearity distortion correction, and registration to the T_1_-weighted image. The spatial transformation from the T_1_-weighted registration was also applied to the diffusion vectors and gradient field tensors. The Oxford Center for fMRI of the Brain's (FMRIB) Software Library (FSL) was used to calculate the diffusion metrics and perform normalization to MNI152 space (13, 14). Fractional anisotropy (FA) maps were generated by fitting diffusion tensors to the processed diffusion MRI dataset registered to the T_1_-weighted structural images using's FSL's dtifit with correction for gradient non-linearities. FSL's tract-based spatial statistics (TBSS) was then used to non-linearly normalize the FA images (FNIRT) to the FMRIB58 FA template in 1 mm^3^ MNI152 space. The white matter features were extracted using Nilearn (version = 0.5.2), an open-source Python module for statistical learning on neuroimaging data (15). Nilearn's NiftiLabelsMasker (resampling target = data) was used to extract the mean fractional anisotropy within 48 white matter regions from The Johns Hopkins University-International Consortium for Brain Mapping's white-matter labels 1 mm^3^ atlas (JHU-ICBM-DTI-81) included with FSL (16), which were used as features in the white matter prediction pipelines (Supplementary Table 2).

### Resting state features

Resting state timeseries were acquired in two sessions with eyes open and relaxed fixation on a crosshair. Within each session, two runs (14 min and 24 s each) were completed with the images acquired with alternated phase encoding directions (i.e., left-right and right-left). If resting state data from two sessions were present, only the data from the first complete session were analyzed. The HCP preprocessing pipeline for resting state fMRI consisted of distortion correction, motion correction, denoising using FMRIB's ICA-based X-noiseifier (FIX), registration to the T_1_-weighted image, and spatial normalization to 2 mm^3^ MNI152 space (17). Nilearn's NiftiLabelsMasker (resampling target = data, spatial smoothing = none, band-pass temporal filter = 0.008–0.100 Hz) was used to extract the mean time series from the regions of the asymmetric bootstrap analysis of stable clusters (BASC) 122 region brain parcellation from the preprocessed resting state images in MNI152 space for each phase encoding direction run (18). We chose the 122 region BASC parcellation because it performed the best of the predefined atlases and almost as well as the best data-driven brain parcellation methods (i.e., Dictionary Learning ℓ_1_) in the recent study by Dadi et al. (19), which completed an exhaustive comparison of 240 resting state fMRI classification prediction pipelines across multiple datasets and conditions. We chose a predefined atlas to increase the efficiency of the analyses as well as the interpretability of the functional connectivity measures, which otherwise vary depending on the sampling (e.g., when using a data driven parcellation method). Tangent pairwise functional connectivity was calculated using Nilearn's ConnectivityMeasure for each phase encoding direction, and then averaged resulting in 7,381 resting state features per connectivity measure (20, 21).

### Motor task features

The task-evoked fMRI experiments followed the resting state fMRI sessions. The motor task was adapted from Buckner et al. and designed to map brain motor areas (22, 23). Each run consisted of alternating 12 s blocks of tapping the left or right fingers, squeezing of the left or right toes, and movement of the tongue. Each movement block was performed twice and preceded by 3 s visual cues. Each run also contained three 15 s fixation blocks for a total of 3 min and 24 s per run. Two motor task runs were performed with the images acquired using alternated phase encoding directions (i.e., left-right and right-left). The HCP preprocessing pipeline for task fMRI consisted of distortion correction, motion correction, registration to the T_1_-weighted image, and spatial normalization to 2 mm^3^ MNI152 space. Statistical maps of the preprocessed images in MNI152 space were generated for each run using FSL's Improved Linear Model (FILM) with prewhitening (24, 25). The functional images were spatially smoothed with a 4 mm^3^ full width half maximum (FWHM) Gaussian smoothing kernel and then high-pass temporally filtered (cutoff = 200 s). The task was modeled using the hemodynamic response function (double-gamma, phase 0 s) convolved vectors for the visual cue and the left fingers, right fingers, left toes, right toes, and tongue movements as explanatory variables. We included the temporal derivatives of the task blocks as covariates of no interest. We then generated average activation maps across the two runs in a second-level fixed effects analysis. The contrast parameter estimates (COPE) for the movement blocks relative to the fixation blocks were extracted voxelwise from the second-level analyses for the left and right finger tapping movements using Nilearn's NiftiMasker and a gray matter mask. The gray matter mask included the cortical gray matter, subcortical nuclei, brainstem, and cerebellum resulting in 194,807 features for each finger tapping movement (i.e., left and right hands), which were used in the motor task prediction pipelines. The left and right finger tapping movements were each assessed separately in their own prediction pipelines.

### Strength

Grip strength from the dominant hand was used as the measure of strength. Strength testing was performed using the National Institutes of Health (NIH) Toolbox Grip Strength Test and measured seated using a Jamar Plus Digital dynamometer with the feet on the ground, the elbow bent at 90°, the arm against the trunk, and the wrist in neutral (26). For each hand, the participant performed a less than full force practice trial followed by a full force trial, in which the participant squeezed as hard as possible for a count of three. We used the raw grip strength values normalized to the entire NIH Toolbox Normative Sample (age ≥18 years) without adjusting for sex or age. A score of 100 indicates performance that was at the national average, and a score of 115 or 85 indicates performance one standard deviation above or below the national average, respectively (27). The grip strength testing protocol has been shown to have good to excellent test-retest reliability and good validity compared to a Biodex System 3 Isokinetic Dynamometer (Biodex Medical Systems, Inc. Shirley, New York, USA).

### Training and testing datasets

From the HCP 1,200 subjects release 3T data, 1,047 participants had complete MRI datasets. A complete dataset included at least one T_1_-weighted structural image, one T_2_-weighted structural image, completion of 50% of the diffusion MRI acquisition, and completion of one set of the left-right and right-left phase encoding runs for both the motor task and resting state fMRI acquisitions. Of the complete MRI datasets, one participant's data were excluded for a missing grip strength score, and another participant was excluded for having an outlier grip strength score of 55.3, which was more than five standard deviations below the HCP's average grip strength score. The final dataset consisted of 1,045 participants (age = 28.7 ± 3.7, 514 females) with an average grip strength score of 116.7 ± 11.2. The final dataset was then split into training and testing datasets. The training dataset was used to train the prediction pipeline, and the testing dataset was used as an independent, holdout dataset to provide an unbiased estimate of the prediction pipeline performance. The HCP 1,200 subjects release 3T dataset contains 143 pairs of monozygotic and 85 pairs of dizygotic twins confirmed by genotyping as well as non-twin siblings. To preserve independence of the training and testing datasets from genetic and environmental factors, the 75 non-related participants from unique families, based on the mother, father, and family identifiers, were assigned to the testing dataset (age = 28.5 ± 3.7 years, 38 females, grip strength score = 117.5 ± 10.4). The remaining 970 participants, which included twin and sibling participants, were assigned to the training dataset (age = 28.8 ± 3.7 years, 525 females, grip strength score = 116.6 ± 11.2). The training and testing datasets did not significantly differ on age (*t* = 0.677, *p* = 0.499), sex (*X*^2^ = 0.335, *p* = 0.563), handedness (*X*^2^ = 2.909, *p* = 0.088), or grip strength (*U* = 34,177.5, *p* = 0.383; Table 1).

**Table 1:** Summary of training and testing datasets.

	**Training (*n* = 970)**	**Testing** **(*n* = 75)**	***p*-Value**
Age (years)	28.8 ± 3.7	28.5 ± 3.7	0.499
Female (*n*)	525	38	0.563
Right-handed (*n*)	885	64	0.088
Grip strength score	116.6 ± 11.2	117.5 ± 10.4	0.383

### Prediction pipelines

Analyses were performed using Scikit-learn (version = 0.21.2), an open-source python package for machine-learning (28). For the first-level modeling, we used linear regression (LinearRegression), which is a statistical method that determines the best linear function for all points (*X, y*) that minimizes the sum of the squared errors *via* ordinary least squares. Because of the small size of the dataset in comparison to the high-dimensional feature space, especially for the resting state and motor task prediction pipelines, the regression model is prone to overfitting. Therefore, we used non-sparse (ℓ2) models to penalize excessive model complexity and encourage generalizability. As the number of features (*p*) for the resting state and motor task fMRI greatly exceeded the number of participants (*n*) (i.e, *p* >> *n*), dimensionality reduction was performed using principal components analysis (PCA). Using PCA, we transformed the features to the set of uncorrelated principal components (*n* – 1), each a linear combination of the original features using singular value decomposition. The features were winsorized (average ± 3 SD) to limit extreme values, and then each feature was mean centered and scaled to unit variance. Winsorizing and scaling were performed prior to PCA, based on the training data, and then used to transform the testing data, which kept the processing independent of the testing data. This was similarly done for PCA and the calculation of the tangent functional connectivity measure, which requires the calculation of a group average covariance matrix. Hyperparameter tuning of the ℓ2 regularization parameter (*C* = 0.00001, 0.0001, 0.001, 0.01, 0.1, 1, 10, 100, 1,000, 10,000, 100,000) was performed *via* nested five-fold cross-validation using grid search and the mean squared error as the performance metric. We then repeated the grid search again using a finer range of regularization parameters.

### Multimodal model

A stacked ensemble of prediction pipelines was used to combine the first-level prediction pipelines for the gray matter, white matter, resting state, and motor task features. The left and right motor task prediction pipelines were included separately. In a standard stacking ensemble, the training dataset is used to fit a first-level model, and then the training dataset predictions from the first-level models are used to train a second-level model. The use of the same training dataset to fit and generate the regressors for the second-level model can lead to overfitting, especially for datasets that have a large number of features relative to the number of samples, which is the case for the resting state and motor tasks. To overcome this barrier, we used a 10-fold cross-validation framework for each first-level prediction pipeline and passed the out-of-fold predictions as regressors to the second-level model (i.e., pre-validation) (29). Linear regression without regularization was used for the second-level multimodal modeling.

### Testing performance

We assessed the performance of the prediction pipelines for each of the first-level models as well as the second-level multimodal model in the independent, holdout testing dataset to provide an unbiased estimate of model of performance and generalizability. Testing performance was assessed using the mean absolute error (MAE), root mean squared error (RMSE), and *R*-squared (*R*^2^) calculated with scikit-learn. The following equation was used for *R*^2^:


$$R^{2}=1-\frac{\sum_{i}{\left(y_{i}-p_{i}\right)}^{2}}{\sum_{i}{\left(y_{i}-\bar{y}\;\right)}^{2}}$$

In this equation, *y*_*i*_ = the measured grip strength scores, *p*_*i*_ = the predicted grip strength scores, and ȳ = the average of the measured grip strength scores. When using a model fit to a training dataset to make a prediction on a testing dataset, *R*^2^ can be negative if the model performs worse than a baseline model that always predicts ȳ. Permutation testing was used to validate the unbiased nature of the prediction pipelines in which the first-level training dataset grip strength scores were randomly permuted prior to entering the prediction pipeline (30). We recorded the MAE, RMSE, and *R*^2^ of the testing dataset predictions for 10,000 permutations of the training dataset to create a null distribution of performance metrics. From the null distribution, we calculated a *p*-value (one-tailed) for the performance of the prediction pipeline fit to the unpermuted training dataset to provide an estimate of statistical significance relative to chance.

### Sex as a confound

Strength was significantly greater in males than females (**Figure 2** and **Table 3**), making sex a potential confound in the analyses, so we then investigated the performance of the prediction pipelines after correcting the brain features and grip strength scores for sex. Sex correction was performed by demeaning each feature and the strength scores using the mean values of the corresponding sex. Demeaning as applied is equivalent to using linear regression to correct for a binary variable. To further investigate strength and sex in brain prediction, we flipped the analyses and explored the power of the brain features to predict sex by using a logistic regression classification model with ℓ2 penalization (LogisticRegression). We performed the feature selection and model training similar to the strength regression prediction pipelines for the first-level models as well as the multimodal classification model (i.e., 10-fold cross-validated stacked classification ensemble). The sex prediction pipeline performance was compared with and without correcting the brain features for grip strength. Strength correction was performed using linear regression and then training on the residuals. For sex prediction, testing performance was assessed using percent accuracy and the area under the receiver operating characteristic curve (AUC) calculated with scikit-learn. The AUC was calculated by plotting the true positive rate (TPR) as a function of the false positive rate (FPR) at varying threshold settings. The strength and sex correction parameters were calculated on the training dataset and then applied to the testing dataset. Finally, we assessed the ability to predict sex using the grip strength measures alone and logistical regression.

### Visualization of first-level prediction pipeline features

To visualize and compare the brain features that made reliable contributions to the prediction of strength and sex, bootstrapping was performed. For each first-level model, *p*-values for the model coefficients were calculated by fitting the models to 10,000 bootstrapped samples from the training dataset (hyperparameter tuning same as that determined from model fit to entire training dataset), then converting the model coefficient to *Z*-values (bootstrapped mean of each coefficient / bootstrapped standard deviation of each coefficient), and finally transforming the *Z*-values to *p*-values using a two-tailed normal distribution. The coefficient *p*-values were corrected with a false discovery rate (FDR) of *q* < 0.05 to correct for multiple comparisons (31). When PCA was employed, the model coefficients for these pipelines were first inverse transformed back to the original feature space before conversion to *p*-values. The significant model coefficients (FDR-corrected) were visualized to assess the contributions of the first-level features to the prediction of strength and sex. We performed the FDR correction using the MNE Python package (version = 0.21.0).

The labeling of the 122 regions in the BASC parcellation, used to calculate functional connectivity, is unordered and does not correspond to any particular resting state functional brain network. To assist in interpretation of the resting state functional networks underlying prediction, each of the 122 regions was assigned to a resting state functional network based on the maximum percent overlap between the region and binary maps of the visual, somatomotor, dorsal attention, ventral attention, limbic, frontoparietal, and default mode networks as well as subcortical and cerebellar networks. The cortical networks were defined using the seven network parcellation developed by Yeo et al. (23). The subcortical network was defined used the Harvard-Oxford subcortical atlas and contained the thalamus, caudate, putamen, globus pallidus, hippocampus, amygdala, and brainstem (11, 32–34). The cerebellar network was defined using the probabilistic human cerebellum atlas developed by Diedrichsen et al. (35). Both the subcortical and cerebellar atlases are included with FSL. The FDR-corrected model coefficients weights for the resting state prediction pipeline were visualized using circular graphs plotting both the positive and negative connections within and between the resting state functional networks using Circos (36). The FDR-corrected white matter and task model coefficients were visualized overlaid the MNI152 T_1_-weighted brain template. Finally, we used Spearman correlations to directly compare the non-corrected model coefficients and quantify the similarity between the strength and sex prediction models.

### Statistical testing

Student *t*-tests and correlation analyses were performed using the SciPy Python package (version = 1.2.1). An α <0.05 was considered statistically significant. Correlation and AUC plots were generated using the Matplotlib Python package (version = 3.1.0).

## Results

**Figure 1:**
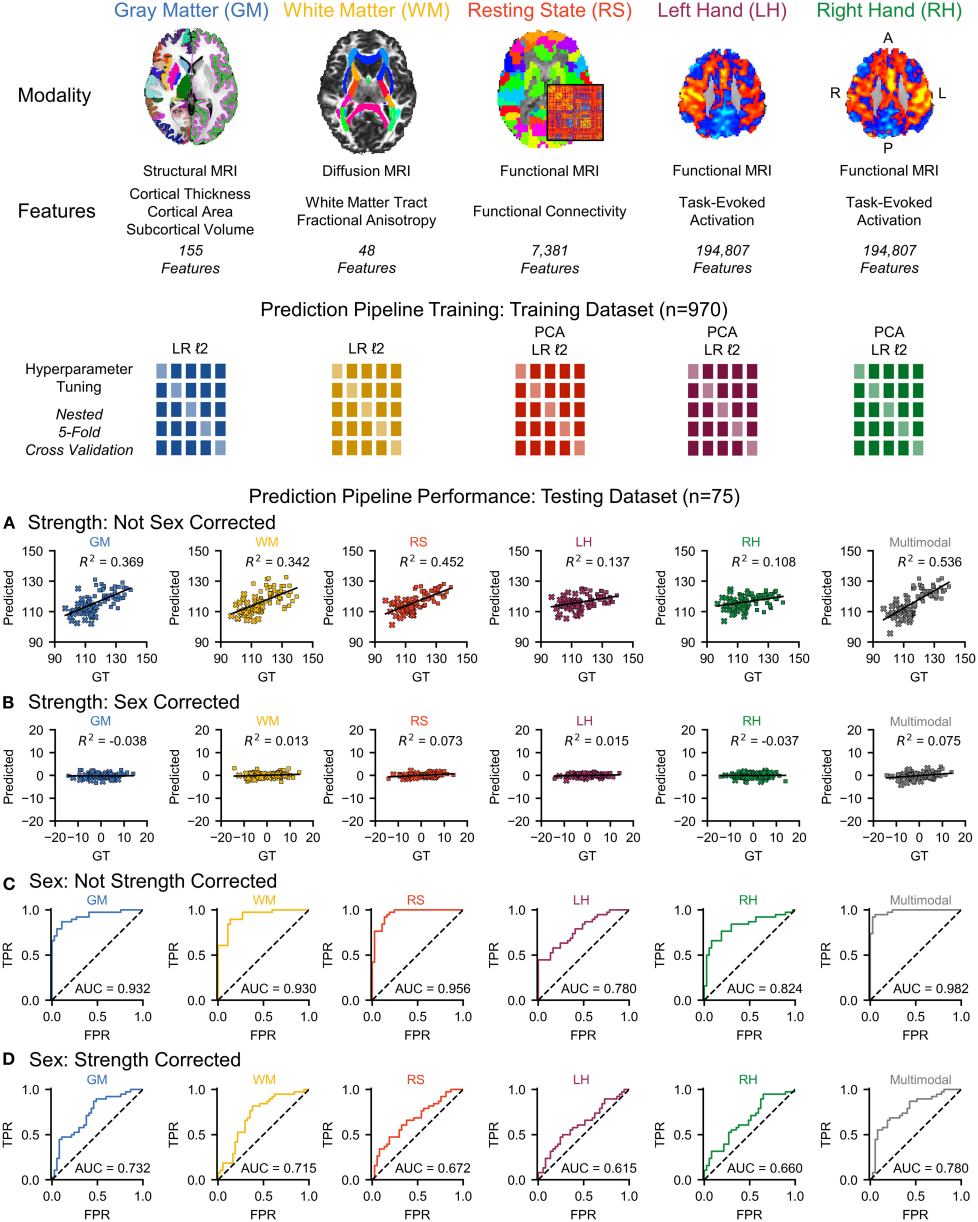
We used multimodal brain MRI and grip strength scores to characterize and quantify the brain's contribution to strength through multivariate predictive modeling. Multimodal brain MRI provides measures on gray matter (GM) morphometry, white matter (WM) fractional anisotropy, resting state (RS) functional connectivity, and left hand (LH) and right hand (RH) motor task activation. We trained a prediction pipeline for each MRI modality on the training dataset (*n* = 970) using non-sparse (ℓ2) linear regression (LR) models with principal component analysis (PCA) for dimensionality reduction, when applied, and nested five-fold cross-validation for hyperparameter tuning. We then used stacked ensembles of the prediction pipelines to combine the first-level models into second-level multimodal models. **(A)** Each MRI modality and a multimodal model significantly predicted strength in an independent testing dataset (*n* = 75). **(B)** However, sex was identified as a potential confound, and correcting for sex substantially reduced the predictive power for each MRI modality and the multimodal model (female = × , male = ■). **(C)** Next, we flipped the analysis and investigated the use of multimodal brain MRI to predict sex. Each of the MRI modalities and a multimodal model significantly predicted sex. **(D)** Correcting the brain features for strength, however, reduced the accuracy of each MRI modality and a multimodal model for predicting sex. GT, ground truth; TPR, true positive rate; FPR, false positive rate; AUC, area under the curve. L, left; R, right; A, anterior; P, posterior.

We assessed the performance of each modality on the independent testing dataset (*n* = 75), providing an unbiased estimate of performance. Each modality had predictive power for strength that exceeded chance accuracy (*p* ≤ 0.001) with the resting state prediction pipeline having the highest performance of the individual modalities, explaining more than 45% of the variance in strength (*R*^2^ = 0.452). The gray matter and white matter prediction pipelines performed comparably to each other, both explaining more than 30% of the variance in strength. The left and right hand prediction pipelines had the lowest performance, each explaining <15% of the variance in strength (Figure 1 and Table 2). The multimodal prediction pipeline outperformed the individual modality prediction pipelines explaining more than 50% of the variance in strength (*R*^2^ = 0.535, MAE = 5.95, and RMSE = 7.09; Figure 1 and Table 2).

**Table 2:** Grip strength prediction testing performance (*n* = 75).

**Pipeline**	**MAE**	***p*-Value**	**RMSE**	***p*-Value**	***R* ^2^**	***p*-Value**
**Not sex corrected**						
Gray matter	6.92	<0.001	8.27	<0.001	0.369	<0.001
White matter	6.41	<0.001	8.44	<0.001	0.342	<0.001
Resting state	6.18	<0.001	7.71	<0.001	0.452	<0.001
Left hand	8.11	0.002	9.67	0.002	0.137	<0.001
Right hand	7.89	0.004	9.83	0.001	0.108	0.001
Multimodal	5.95	<0.001	7.09	<0.001	0.536	<0.001
Sex corrected*						
Gray matter	5.20	0.126	6.04	0.014	−0.038	0.014
White matter	4.89	0.087	5.89	0.066	0.013	0.069
Resting state	4.82	0.005	5.71	<0.001	0.073	<0.001
Left hand	4.99	0.026	5.89	0.003	0.015	0.003
Right hand	5.07	0.071	6.04	0.018	−0.037	0.018
Multimodal	4.78	0.001	5.71	<0.001	0.075	<0.001

*Testing performance assessed using grip strength scores corrected for sex.

**Figure 2:**
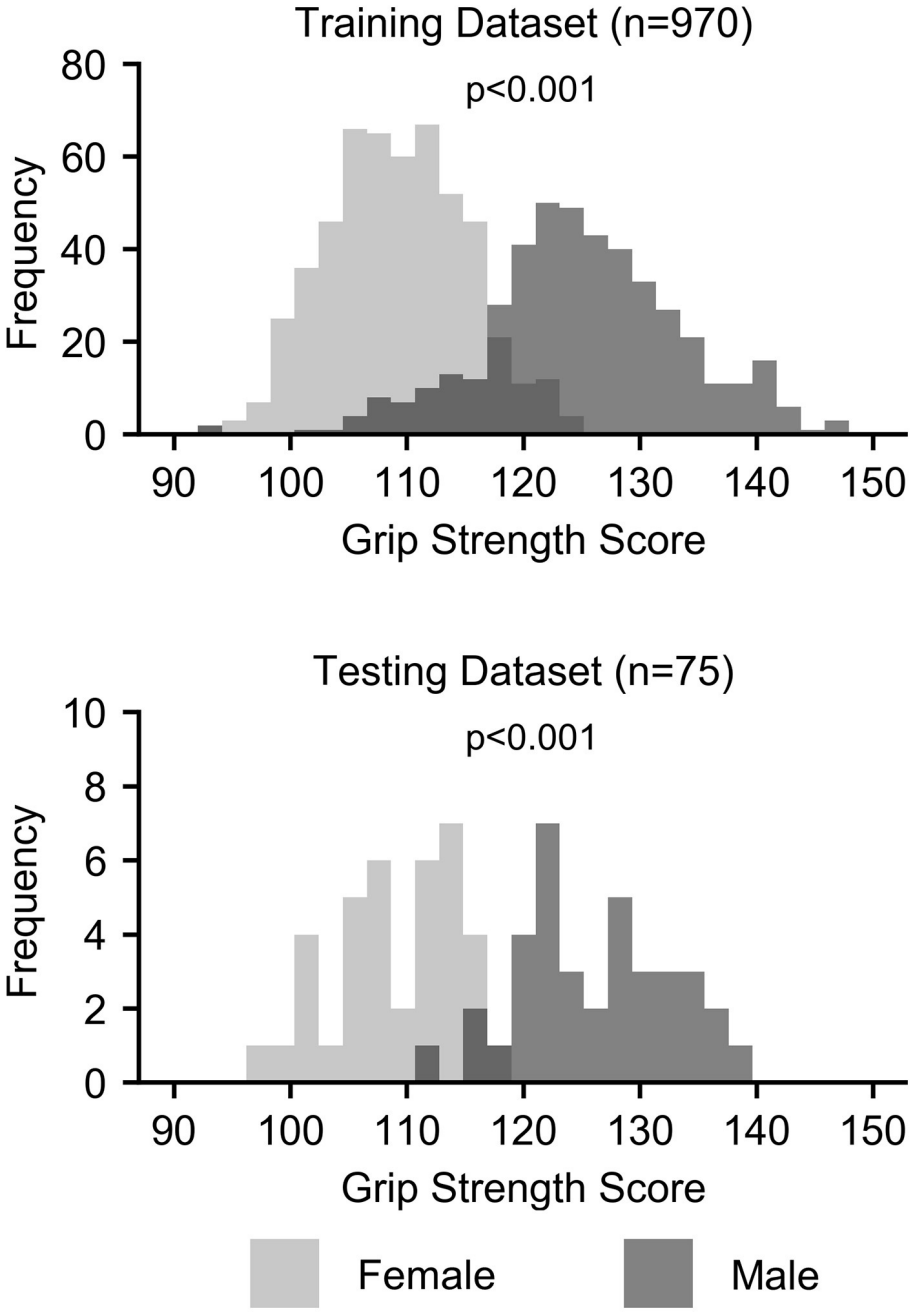
Histograms of grip strength scores by sex for the training and testing datasets.

Grip strength, however, was significantly higher in males than in females in both the training (*p* < 0.001) and testing (*p* < 0.001) datasets, and sex was identified as a potential confound (Figure 2 and Table 3). After correcting for sex, the predictive power was substantially reduced for each first-level model (Figure 1 and Table 2). While the resting state prediction pipeline continued to have the highest performance of the individual modalities, resting state functional connectivity only explained slightly more than 7% of the variance in strength after correcting for sex (*R*^2^ = 0.073, *p* < 0.001). The performance of the second-level multimodal prediction pipeline was likewise reduced with similar performance as the resting state prediction pipeline (*R*^2^ = 0.075, *p* < 0.001).

**Table 3:** Grip strength scores by sex.

	**Female**	**Male**	**Cohen's *d***	***p*-Value**
Training (*n* = 970)	109.1 ± 6.0	125.5 ± 9.1	2.1	<0.001
Testing (*n* = 75)	109.0 ± 5.2	126.1 ± 6.6	2.9	<0.001

Next, we flipped the analysis and investigated the use of multimodal brain MRI to predict sex. Each of the MRI modalities significantly predicted sex (*p* < 0.001). The resting state prediction pipeline had the highest performance of the individual modalities, predicting sex with 89.3% accuracy (AUC = 0.956). The gray matter and white matter prediction pipelines performed comparably to each other, both having accuracies > 80%. The left and right hand prediction pipelines had the lowest performance, each with accuracies lower than 80%. The second-level multimodal prediction pipeline had an accuracy of 93.3% (AUC = 0.982; Figure 1 and Table 4). Correcting the brain features for strength, however, reduced the accuracy of predicting sex for each modality, the resting state prediction pipeline had the lowest accuracy of the individual modalities (accuracy = 57.3%, AUC = 0.672). The multimodal prediction accuracy dropped to <70% after correction for strength (AUC = 0.780; Figure 1 and Table 4). In comparison, using grip strength alone predicted sex with 94.7% accuracy (AUC = 0.985, *p* < 0.001).

**Table 4:** Sex prediction testing performance (*n* = 75).

**Pipeline**	**Accuracy (%)**	***p*-Value**	**AUC**	***p*-Value**
**Not strength corrected**
Gray matter	84.0	<0.001	0.932	<0.001
White matter	85.3	<0.001	0.930	<0.001
Resting state	89.3	<0.001	0.956	<0.001
Left hand	68.0	0.005	0.780	<0.001
Right hand	78.7	<0.001	0.824	<0.001
Multimodal	93.3	<0.001	0.982	<0.001
**Strength corrected**
Gray matter	68.0	0.001	0.732	<0.001
White matter	69.3	<0.001	0.715	<0.001
Resting state	57.3	0.121	0.672	0.005
Left hand	58.7	0.057	0.615	0.051
Right hand	61.3	0.043	0.660	0.015
Multimodal	69.3	<0.001	0.780	<0.001

**Figure 3:**
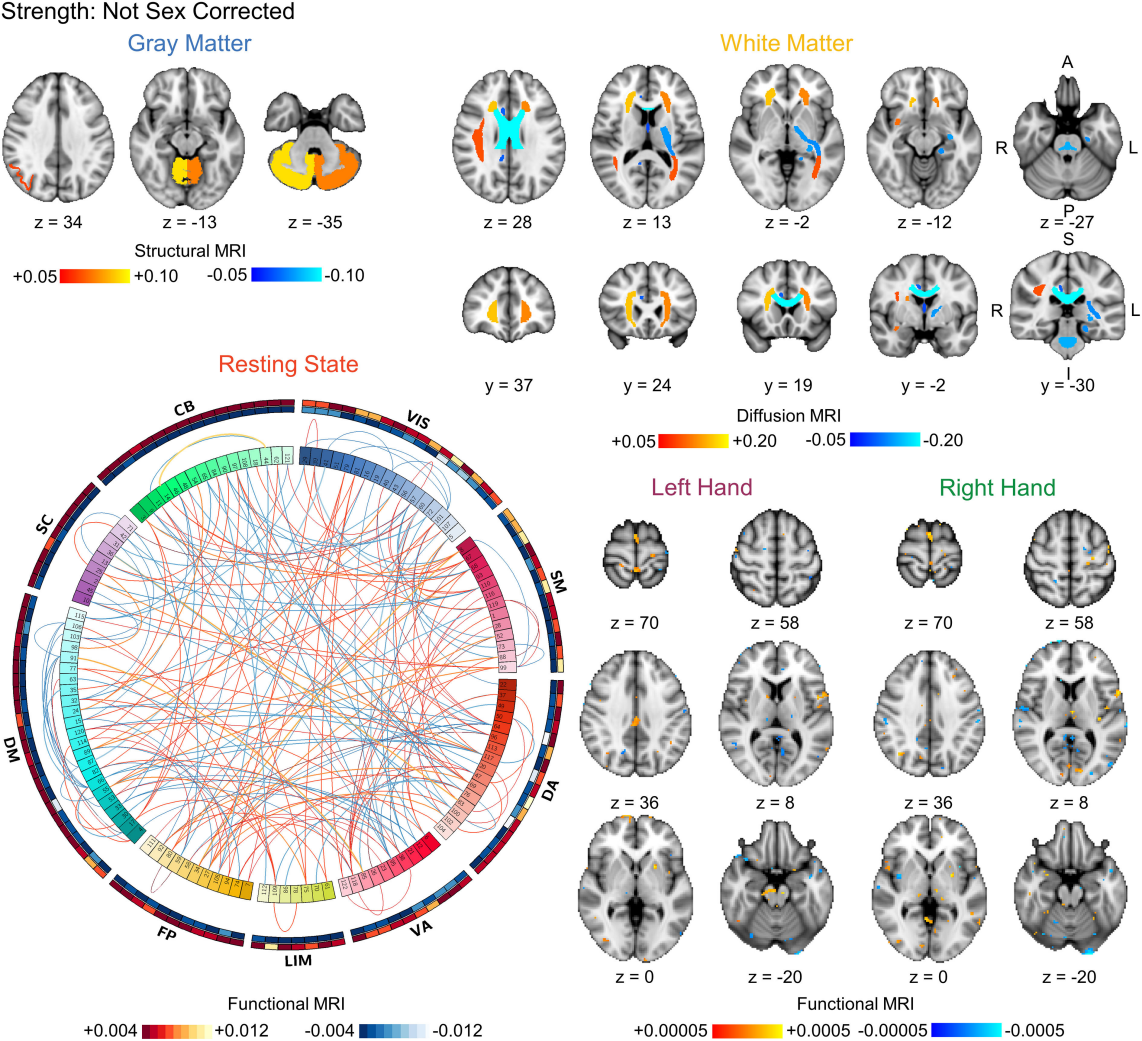
Visualization of the bootstrapped FDR-corrected (*q* < 0.05) first-level model coefficients of each MRI modality for strength prediction without correction for sex. Note the similarity between these models and the sex prediction models without correction for strength shown in Figure 4. The outer bands in the resting state connectivity graph show the sum of the positive and negative connections passing FDR-correction. VIS, visual; SM, somatomotor; DA, dorsal attention; VA, ventral attention; LIM, limbic; FP, frontoparietal; DM, default mode; SC, subcortical; CB, cerebellar. L, left; R, right; A, anterior; P, posterior; S, superior; I, inferior.

**Figure 4:**
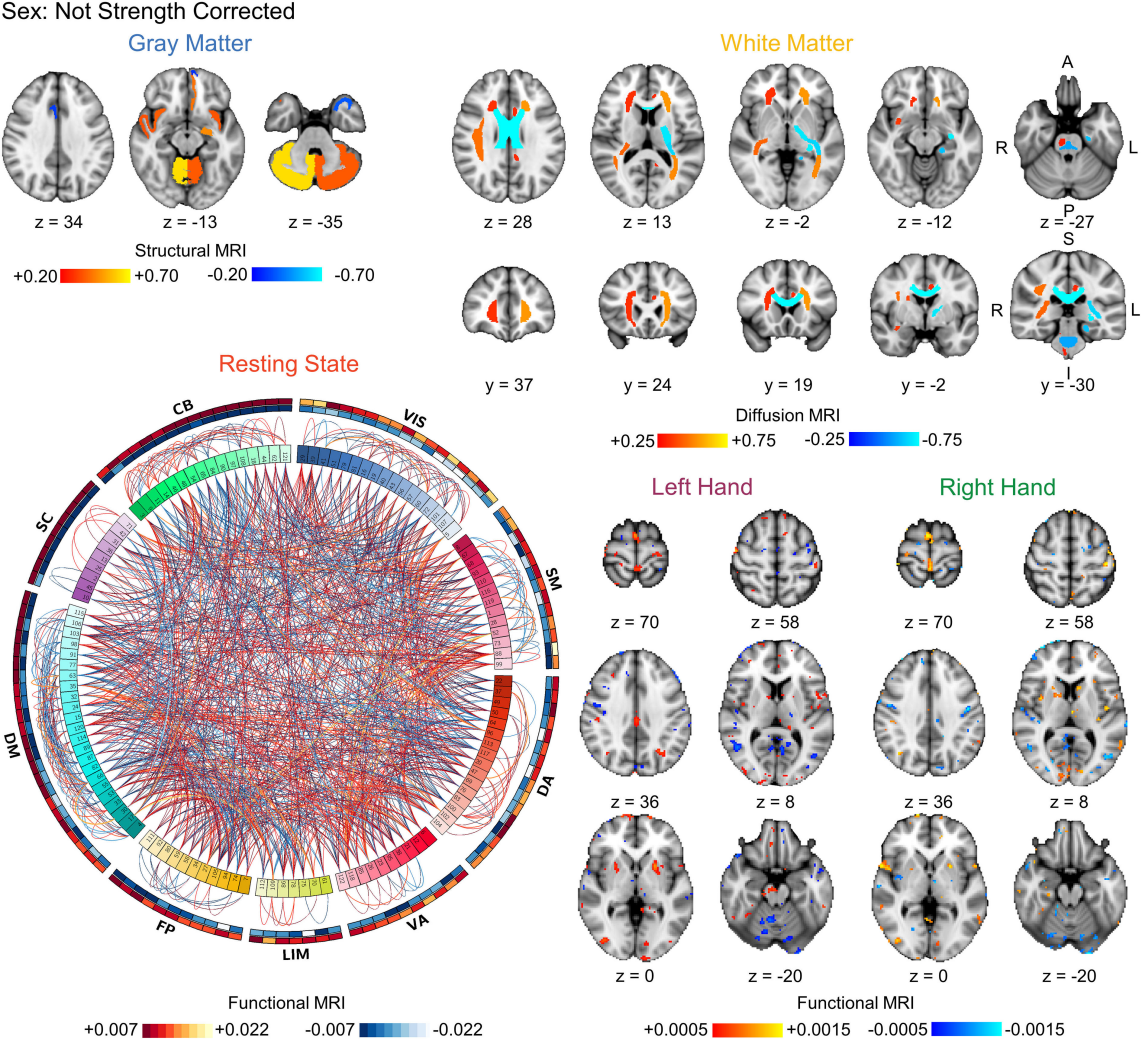
Visualization of the bootstrapped FDR-corrected (*q* < 0.05) first-level model coefficients of each MRI modality for sex prediction without correction for strength. Note the similarity between these models and the strength prediction models without correction for sex shown in Figure 3. The outer bands in the resting state connectivity graph show the sum of the positive and negative connections passing FDR-correction. VIS, visual; SM, somatomotor; DA, dorsal attention; VA, ventral attention; LIM, limbic; FP, frontoparietal; DM, default mode; SC, subcortical; CB, cerebellar. L, left; R, right; A, anterior; P, posterior; S, superior; I, inferior.

Finally, we compared the coefficients between the strength prediction and sex prediction models without correction for sex or strength, respectively, to assess the similarity in the brain features driving the predictions. The FDR-corrected coefficient maps between the strength and sex prediction show substantial overlap for each modality, indicating that both models are using similar information in their predictions (Figures 3, 4). To quantify the level of similarity, we used Spearman correlations to directly compare the model coefficients between the strength and sex prediction models. The model coefficients were moderately to strongly correlations with the white matter models having the greatest correlation (ρ = 0.762, *p* <0.001) and the resting state models having the lowest correlation (ρ = 0.482, *p* <0.001), further demonstrating a substantial but not complete degree of similarity in the information underlying the prediction of strength and sex (Table 5).

**Table 5:** Correlation between strength and sex prediction model coefficients^*^.

**Pipeline**	**ρ**	***p*-Value**
Gray matter	0.653	<0.001
White matter	0.762	<0.001
Resting state	0.482	<0.001
Left hand	0.686	<0.001
Right hand	0.689	<0.001

*Models not corrected for sex or strength, respectively.

## Discussion

We initially sought to characterize and quantify the brain's contribution to strength by developing multimodal brain-based MRI pipelines to predict grip strength. Sex, however, was identified as a clear confound in the analyses, complicating the prediction of strength. Each MRI modality and a multimodal prediction pipeline could accurately predict strength; however, correcting for sex substantially reduced the accuracy. When flipping the analyses and classifying sex from brain MRI, each MRI modality and a multimodal prediction pipeline could accurately predict sex; however, correcting the brain features for strength reduced the accuracy of predicting sex. Comparing the coefficients between the models, we identified substantial but not complete similarities between the brain features driving the prediction of strength and sex. As demonstrated by our results, the interpretation of brain-based prediction models can be problematic in the presence of confounds: are the models predicting strength or sex? In the following, we discuss confounds in brain decoding analyses, their implications in the development of brain-based biomarkers, and possible strategies to overcome these challenges and improve interpretation.

Confounds are a well-known issue in statistical modeling and machine-learning applications (5–7). Confounds create ambiguity in the source of the information driving the prediction and uncertainty in the measure being predicted. In other words, confounds affect the relationship between the features and the predicted measure. In the case of strength prediction, sex is a clear confound in this sample. While males in this sample have only ≈ 15% greater grip strength than females on average, the distribution of strength is clearly bimodal, with highly significant differences in the distribution of strength between the sexes (Cohen's *d* > 2.0). In fact, grip strength alone predicted sex with > 90% test accuracy. Considering the substantial reduction in strength prediction after correcting for sex, the ability to predict sex with high accuracy using the multimodal brain features, and the similarity between the strength and sex prediction models, the strength prediction model, at least in part, is likely learning patterns of features associated with sex. If the accuracy of strength prediction was the only aim, then sex being a confound is less of a concern. However, the initial goal of this study was to uncover the brain's contribution to strength to develop models that may be used as brain-based biomarkers in conditions affecting strength. We are not interested in using features for strength prediction that are related to sex but invariant to strength. Therefore, for the intended application, the interpretation of the models becomes as important as accuracy.

Sex is a recognized confound in neuroimaging. Males on average have larger total brain size than females (37). In addition to global differences in brain size, localized alterations in cortical thickness and subcortical volumes have also been identified between sexes, and gray matter morphometry has been used to predict sex as well as white matter characteristics and both resting state and task-evoked fMRI measures (38–43). Recently, sex differences in brain-based biomarkers of intelligence have been uncovered, suggesting that the generation of intelligence in males and females may utilize distinct brain networks (44). Debate exists regarding the interpretation, meaning, and importance of sex differences in the brain (45–48). A recent quantitative synthesis by Eliot et al. (49) challenges the importance of sex differences in the human brain, arguing that many structural and functional sex differences identified with MRI are largely negligible after correcting for brain size. A more recent large-scale brain-based MRI study from the UK Biobank with more than 40,000 participants uncovered sex differences in two-thirds of the gray matter brain measures investigated, which were independent of brain size when accounting for non-linear relationships between local brain region morphometry and brain size (50). While the effect of sex was small after correction, structural and functional differences in the brain, even if small, could have meaningful consequences on brain function, especially in aggregate (51, 52). Overall, the meaning of these sex differences and their role in human behavior, mental health, and the brain in health and disease remain to be uncovered. When taken together and with respect to the present findings, identifying and interpreting sex differences in the brain is complex, especially when considering sex-correlated confounds such as strength and brain size.

Age is another important confound in neuroimaging. The brain is not a static organ but changes over the lifespan (50). Similarly to sex prediction, gray matter, white matter, and resting state and task-evoked fMRI brain measures can predict age (53–58). The aging, cognition, and dementia fields have made substantial progress in developing biomarkers of normal and pathological age-related changes in the brain. The difference between predicted and actual brain age, the brain age gap, is the most commonly used measure, providing a single metric to assess brain health with respect to normal aging (59). Brain age biomarkers are already showing potential diagnostic and prognostic value in several conditions including schizophrenia and Alzheimer's disease (60–65). Disease-related processes as well as genetic, lifestyle, and health factors are thought to affect brain aging, but the impact of these factors are still unclear (See Wrigglesworth et al. (66) for review). Sex differences in the brain that accompany aging have also been identified (67–69). A recent study demonstrated differences in the predicted age gap between males and females with a family history of Alzheimer's disease. Sex, *APOE* genotype status, and physical activity demonstrated a significant interaction. Males with +*APOE4* genotype who engage in physical activity had younger predicted brain age than the corresponding females, suggesting that physical activity influences brain aging differentially in +*APOE4* males and females (70). In the prediction of age, sex is not likely a significant confound that impacts interpretability as long as sex is balanced across the age groups. However, if the older participants were disproportionately male and the younger participants were disproportionately female, sex would be a confound, similar to strength in the present study. Sex differences in brain structure and function over the lifespan could impact the accuracy of the age prediction models and substantiate correcting the features for sex or modeling each sex separately. In regards to age as a confound in the prediction of strength, participants in the HCP were healthy young adults (age = 22–37 years), so we were not concerned about age being an important confound. Strength, however, on average declines with age (71–73), and even within the HCP dataset, grip strength was weakly negatively correlated with age (*n* = 1,045, *r* = −0.104, *p* < 0.001). If we were developing predictive models of strength across a broader age range, age would become a confound in the analyses making it difficult to determine whether we are predicting strength or age.

So far, we have discussed participant-related confounds of sex and age and their potential confounding effects in brain-based predictions. Additional participant-related confounds include anatomical variability (e.g., brain size and shape), arousal level (e.g., sleep and caffeine use), mental and emotional state, head movement (i.e., ability to stay still), and overall health status (e.g. presence of medical conditions, medication use, and comorbidities) (74). Procedure-related confounds occur from factors within the study design that can influence the participant measures and include experimental instructions (e.g., eyes open or closed in resting state fMRI), time of day, auditory and visual noise, and image acquisition (e.g., imaging parameters, software, and hardware) and processing methods (e.g., spatial normalization methods) (75–77). Brain shape and size are known to vary across populations, change with age, and be affected by diseases (78–82). For example, ventricular enlargement and cortical atrophy often present in elderly patients, and standard spatial normalization algorithms may not be adequate for this population (83). This could lead to age-related biases in the measurements of cortical structures. A model that predicts age using the measurement error vs. actual age-related neurophysiological changes may accurately predict age, but the model would not likely be a strong candidate biomarker. Additionally, even when developing a brain-based diagnostic marker between participants with a specific condition and an age- and sex-matched group of healthy controls, other confounds may still be present. For example, in chronic pain, comorbidities such as anxiety and depression are often present, and the patients may be taking medications for their conditions (84). The presence of comorbidities and medication use could be considered confounds in the development of chronic pain markers as each of the three factors (chronic pain, comorbidities, and medication use) may differ from the control group. Similar issues can arise when combining datasets across multiple sites, where site could be a confound (77, 85). Overall, if any factors vary with respect to the predicted measure, these factors may be potential confounds. The degree of concern regarding these factors will depend on the goal of the biomarker and may require additional interrogation to interpret the features driving the prediction and further validate the model (86). As an example, Liem et al. (53) were concerned that head motion may confound age prediction because head motion affects brain imaging and motion may be higher in older participants. Therefore, the authors included additional analyses to be confident that motion was not driving the prediction of age (53).

In conventional univariate analyses, controlling for confounds can be done statistically by including them as covariates in the model. For example, motion regressors are commonly modeled as covariates when generating subject-level task-evoked fMRI activation maps to account for signal correlated with subject motion, and for group-level fMRI analyses, sex and age can be included to remove their effects when comparing activity between groups. Including confounds as features in decoding models, however, would be counterproductive because confounds contain information that by definition we do not want to use in prediction, so alternative strategies need to be employed. With sampling-based approaches you train models on subsamples of the dataset in which the sampling removes the correlation between the confound and predicted measure and the overall effect of the confound (7). For example, you could match males and females on strength, and only include those matched pairs in the training dataset. This would eliminate the sex differences in strength. However, in the case of strength with strong sex differences, a large portion of the data at the high and low strength levels would be discarded using sampling-based approaches, reducing power and also limiting the domain and range on which the model was trained. Sampling-based approaches can be performed within a cross-validated framework using a stratification factor (e.g., StratifiedKFold). While typically performed *post hoc*, controlling for confounds can be done *a priori as* done in prospective trials where groups are commonly matched on age and sex at enrollment in a study.

While sampling-based approaches may be feasible for one and maybe two confounds, accounting for multiple confounds becomes increasingly complicated and costly (i.e., dropping of unmatched data) and is largely not feasible. Regression-based approaches statistically remove the variance attributed to the confounds from the dataset by fitting a model containing the confound variables to the dataset (7). The modeling is then performed on the residuals, in which the confounding signal has been removed. Regression-based approaches were used in the current study to correct for sex and grip strength. After correcting for sex, only the resting state and the multimodal strength prediction models significantly predicted strength better than a random model. A drawback to regression-based approaches is that they increase the complexity of the prediction pipeline in that the regression-based confound correction model is another step within the prediction pipeline with its own parameters. Another recent alternative proposed by Zhao et al. (87) uses a deep-learning approach with generative adversarial networks that models confounding effect in the feature-learning process such that the model learns patterns of features that are invariant to confounds. The authors validate their method with classification and regression models with continuous and categorical confounds, and the code is openly available on GitHub (https://github.com/qingyuzhao/br-net) (87).

Inherent to our goal of developing brain-based biomarkers of strength is that we need the models to be based on features that underlie the production of force as we want the predictions to be sensitive to changes in strength over time. Sex is a non-modifiable factor, and a strength model based on sex-correlated features invariant of strength would not likely be sensitive to changes in strength. As described and demonstrated, sex is a major confound in the prediction of strength. When accounting for sex, the accuracy for predicting strength from multimodal brain MRI is largely lost. As discussed, males have larger brains than females on average, and the magnitude of the sex differences decreases after correcting for brain size. Sex and brain size are not the only factors that covary. Strength and muscle mass also strongly covary with sex and brain size (88). Strength, muscle mass, and brain size are known to decrease in older age (71–73, 89, 90). Also, physical exercise can decrease age-related losses in the brain's gray and white matter (91–93). While less studied than in the brain and human studies are lacking, sex differences may also present in the spinal cord. Male rats have larger spinal cords by weight and a greater number of motoneurons (94) as well as differences in skeletal muscle fiber morphometry, spindle density, and innervation (95, 96). When taken together, it is not too great of a leap to suggest that a component of the neuromuscular system, such as skeletal muscle mass, could mediate the relationship between sex and brain size. The larger brain size in males could be to support the larger skeletal muscle mass (97).

The cross-sectional nature of the HCP limits the current study to (repeat imaging is only available in a small subset of the dataset, *n* < 50) associations between the brain imaging features and strength. Longitudinal studies with a strength training exercise intervention could determine whether the strength prediction pipelines track increases in strength. If so, this would indicate that the strength prediction models are relying on some information related to strength and provide evidence that the brain features predictive of strength are causally linked to force production. If not, then the models may be relying more on sex-correlated features for the prediction of strength. Longitudinal studies employing within subject models can also help address the issue of confounds allowing us to develop models that predict change in strength from the changes in brain features. A similar argument supporting the use of longitudinal studies has been made by Vidal-Pineiro et al. (98) in the age-prediction field where models developed from cross-sectional studies did not predict longitudinal changes in brain age. The authors suggest that brain age may relate more to early-life factors than longitudinal brain changes, leading to the recommendation that future studies use longitudinal designs when predicting individual changes in brain age is the goal.

Additional improvements can also be made from the current study. The fMRI experiment used to map task-evoked left- and right-hand motor activity was a finger tapping task and not designed to capture signals related to strength. An experiment tuned to force generation may have greater predictive power (99). Similarly, a more diverse sample with greater variation in strength should improve our ability to predict strength. While the use of HCP dataset provided a large, homogenous dataset for this study, future work should include data across sites with varying imaging equipment, imaging parameters, and sample characteristics to generate models that generalize across sites and to the population at large.

The application of multivariate predictive modeling to neuroimaging is increasing our ability to extract clinically relevant information from the brain and make predictions across individuals. While brain-based predictive models have great potential as biomarkers for neurological, neuromuscular, and musculoskeletal conditions, the presence of confounds creates ambiguity in the source of the information driving the prediction and the interpretation of the measure being predicted, decreasing their potential clinical utility. Here we focused on the effects of sex-correlated confounds in brain-based predictive modeling across multiple MRI modalities for both regression and classification models. In addition to sex, other patient-related and procedural confounds are well-known in neuroimaging. Methods to better assess the influence of these confounds on the predicted measure and the development of strategies to mitigate the effects of confounds will increase the interpretability and validity of brain-based biomarkers and further promote their translation to clinical practice.

## Data availability statement

WU-Minn Human Connectome Project (HCP) 1200 subjects release dataset is available from the HCP (www.humanconnectome.org). The use of family tree structure, exact age, and handedness required access to the restricted HCP dataset, which includes potentially identifiable information, and acceptance of the HCP's restricted data use terms. The codes for the HCP preprocessing pipeline and preprocessed datasets are available through HCP. The analysis and prediction pipelines were developed using open-source, commercially usable Python packages (Nilearn, sci-kit Learn, MNE, and SciPy).

## Ethics statement

Ethical review and approval was not required for the study on human participants in accordance with the local legislation and institutional requirements. Written informed consent for participation was not required for this study in accordance with the national legislation and the institutional requirements.

## Author contributions

KW: conceptualization. KW, CL, and SM: data curation. KW, TW, CL, NP, YA, GG, SD, GHG, TH, and SM: methodology. KW, ZT, TW, NP, YA, GG, SD, GHG, TH, and SM: investigation. KW and ZT: formal analysis. KW, ZT, and SM: writing—original draft. KW, ZT, TW, CL, NP, YA, GG, SD, GHG, TH, and SM: writing—review and editing. KW and SM: funding acquisition. All authors contributed to the article and approved the submitted version.
