# Supplementary material for: Confounds in neuroimaging: A clear case of sex as a confound in brain-based prediction
Source: Front Neurol. 2022 Dec 19;13:960760. doi: 10.3389/fneur.2022.960760 (PMC9806266; doi:10.3389/fneur.2022.960760)
Supplement: Supplementary file 1 [file Table_1.DOCX]

Supplementary Material

# Supplementary Tables

**Sup. Table 1. Gray Matter Regions**

| FreeSurfer Gray Matter Regions | |
| --- | --- |
| Location | Region |
| Cortical | Left and Right Banks of the Superior Temporal Sulcus |
|  | Left and Right Caudal Anterior Cingulate |
|  | Left and Right Caudal Middle Frontal |
|  | Left and Right Cuneus |
|  | Left and Right Entorhinal |
|  | Left and Right Fusiform |
|  | Left and Right Inferior Parietal |
|  | Left and Right Inferior Temporal |
|  | Left and Right Isthmus Cingulate |
|  | Left and Right Lateral Occipital |
|  | Left and Right Lateral Orbitofrontal |
|  | Left and Right Lingual |
|  | Left and Right Medial Orbitofrontal |
|  | Left and Right Middle Temporal |
|  | Left and Right Parahippocampal |
|  | Left and Right Paracentral |
|  | Left and Right Pars Opercularis |
|  | Left and Right Pars Orbitalis |
|  | Left and Right Pars Triangularis |
|  | Left and Right Pericalcarine |
|  | Left and Right Postcentral |
|  | Left and Right Posterior Cingulate |
|  | Left and Right Precentral |
|  | Left and Right Precuneus |
|  | Left and Right Rostral Anterior Cingulate |
|  | Left and Right Rostral Middle Frontal |
|  | Left and Right Superior Frontal |
|  | Left and Right Superior Parietal |
|  | Left and Right Superior Temporal |
|  | Left and Right Supramarginal |
|  | Left and Right Frontal Pole |
|  | Left and Right Temporal Pole |
|  | Left and Right Transverse Temporal |
|  | Left and Right Insula |
| Subcortical | Left and Right Cerebellum Cortex |
|  | Left and Right Thalamus Proper |
|  | Left and Right Caudate |
|  | Left and Right Putamen |
|  | Left and Right Pallidum |
|  | Left and Right Hippocampus |
|  | Left and Right Amygdala |
|  | Left and Right Accumbens Area |
|  | Left and Right Ventral Diencephalon |
|  | Brainstem |

**Sup. Table 2. White Matter Regions**

| JHU-ICBM-DTI-81 White Matter Regions |
| --- |
| Left and Right Anterior Corona Radiata |
| Left and Right Anterior Limb of Internal Capsule |
| Body of Corpus Callosum |
| Left and Right Cerebral Peduncle |
| Left and Right Cingulum |
| Left and Right Cingulum of Hippocampus |
| Left and Right Corticospinal Tract |
| Left and Right External Capsule |
| Fornix |
| Left and Right Fornix Stria Terminalis |
| Genu of Corpus Callosum |
| Left and Right Inferior Cerebellar Peduncle |
| Left and Right Medial Lemniscus |
| Middle Cerebellar Peduncle |
| Pontine Crossing Tract |
| Left and Right Posterior Corona Radiata |
| Left and Right Posterior Limb of Internal Capsule |
| Left and Right Posterior Thalamic Radiation |
| Left and Right Retrolenticular Part of Internal Capsule |
| Left and Right Sagittal Stratum |
| Splenium of Corpus Callosum |
| Left and Right Superior Cerebellar Peduncle |
| Left and Right Superior Corona Radiata |
| Left and Right Superior Fronto-occipital Fasciculus |
| Left and Right Superior Longitudinal Fasciculus |
| Left and Right Tapetum |
| Left and Right Uncinate Fasciculus |
